# Tumor characteristics and treatment outcomes of older patients with breast cancer in Jordan

**DOI:** 10.1186/s12905-020-00981-z

**Published:** 2020-06-05

**Authors:** Hikmat Abdel-Razeq, Fadwa Abdel Rahman, Hanan Almasri, Hazem Abdulelah, Mahmoud Abunasser, Mourad Salam, Ayat Taqash

**Affiliations:** 1 Department of Internal Medicine, King Hussein Cancer Center, Queen Rania Al Abdullah Street, P.O. Box: 1269, Amman, 11941 Jordan; 2 School of Medicine, University of Jordan, Amman, Jordan; 3 Department of Radiation Oncology, King Hussein Cancer Center, Amman, Jordan; 4 Office of Scientific Affairs and Research, Amman, Jordan

**Keywords:** Breast cancer, Older women, Jordan, Comorbidities

## Abstract

**Background:**

Less than 10% of newly diagnosed breast cancer cases in Jordan are diagnosed in women 70 years or older. Treatment plans of such patients is less clear and could result in poor outcomes. In this paper, we describe clinical presentation, tumor characteristics and treatment outcomes in this population of breast cancer patients.

**Methods:**

Consecutive patients aged 65 years or older with pathologically-confirmed diagnosis of breast cancer were included. Medical records and hospital databases were searched for patients’ characteristics and treatment outcomes.

**Results:**

A total of 553 patients, mean age ± SD (71 ± 5.1) years, were included. On presentation, 114 (20.6%) patients had metastatic disease and was mostly visceral (81; 71.1%). Patients with non-metastatic disease had poor pathological features including node-positive in 244 (55.6%), high grade (grade III) in 170 (38.7%) and lymphovascular invasion in 173 (39.4%). Patients were treated less aggressively; 144 (32.8%) patients with early-stage disease and 98 (86.0%) with metastatic disease never had chemotherapy.

After a median follow up of 45 months, 5-year overall survival for the whole group was 67.6%. Survival was better for patients with non-metastatic disease (78.8% vs. 25.4%, *P* < 0.001) and for those with node-negative compared to node-positive disease (85.4% vs. 74.1%, *P* = 0.002). On Cox regression, only positive lymph nodes were associated with poor outcome in patients with non-metastatic disease (Hazard Ratio [HR], 1.75; 95% CI: 1.006–3.034, *P* = 0.048).

**Conclusions:**

Older Jordanian patients with breast cancer present with more aggressive features and advanced-stage disease that reflect poorly on treatment outcomes. Older patients were treated less aggressively with less than a third received any chemotherapy.

## Background

Accounting for almost 40% of all cancer cases, breast cancer is the most common cancer in Jordan and its incidence increases with age [1]. Less than 10% of newly diagnosed breast cancer in the Eastern Mediterranean Region, compared to 30% in Western societies, are diagnosed in women 70 years or older [2]. This group of older breast cancer patients remains underrepresented in clinical trials [3], their treatment plan is less clear and have poor outcomes [4, 5].

Pathological features and clinical presentation among older patients with breast cancer are not the same as younger ones. With aging, the percentage of human epidermal growth factor receptor 2 (HER-2) positive disease decreases while estrogen receptors (ER) and progesterone receptors (PR)-positivity increases [6]. Such features, though implicate a better prognosis, are not reflected in real clinical outcomes.

Many previously published studies have shown that older patients are more likely to receive non- standardized care and usually depends more on physician’s preference [7–11]. Compliance to planned treatment is always an issue with increasing age [12].

The poor outcome observed among older patients can also be attributed to comorbidities and its associated medications. Such comorbidities have the potential to affect the mortality of older women regardless of their breast cancer or its treatment [13]. Women with early-stage breast cancer and comorbid conditions, are likely to die from causes other than breast cancer. In one study, using Surveillance, Epidemiology, and End Results (SEER)-Medicare database, inpatient, outpatient, and physician visits were searched to determine the presence of 13 comorbid conditions present at the time of diagnosis. These comorbid conditions include cerebrovascular disease, paralysis, dementia, chronic obstructive pulmonary disease, chronic renal failure, myocardial infarction, congestive heart failure, peripheral vascular disease, diabetes, liver disease, previous cancer, rheumatoid arthritis, and ulcers. A total of 64,034 patients with breast cancer diagnosed at a median age of 75 years were included. The 13 individual comorbid conditions were associated with decreased overall survival and increased mortality [14].

To date, the consequences of treatment disparities, particularly the under treatment of the older patients, have been poorly assessed in population like ours. In this paper, we describe clinical presentations, tumor characteristics, treatment modalities and outcomes among older Jordanian patients with breast cancer.

## Methods

All patients ≥65 year with pathologically-confirmed breast cancer diagnosed between 2006 and 2018, who had all their cancer treatment and follow up at our institution were included. Only patients with invasive carcinoma were included. Medical records and hospital databases were searched for patients’ characteristics and treatment outcomes. All non-Jordanians, and patients with no appropriate follow up were excluded. Data, including detailed pathological features, tumor stage, type of surgery, systemic chemotherapy, radiation therapy, tumor recurrence, and death, were collected through chart review. Data related to tumor size, histological type, lymph node status and the number of metastatic lymph nodes, were obtained directly from the pathology reports. All pathology specimens were reviewed and diagnoses were confirmed at our institution. Estrogen or progesterone receptors were defined as positive if tumor cell nuclei staining is ≥1%. HER-2 was tested using immune histochemical staining (IHC) and tumor cells were considered negative with scores of 0 or + 1 and positive for those with + 3 scores. Samples with + 2 scores were considered equivocal, for which fluorescence in situ hybridization (FISH) was performed. Vital status and death dates were confirmed using our local cancer registry database and the national civil department database. Patients were treated on unified institutional clinical practice guidelines based on standard international ones. Treatment plan was approved by a regularly-conducted weekly multidisciplinary meeting. Because of the retrospective nature of the study and the lack of personal or clinical details of participants that compromise anonymity, consent was waived and the study was approved by King Hussein Cancer Center Institutional Review Board (IRB).

## Statistical analysis

Survival duration was calculated from the date of diagnosis until the date of death or last clinical follow- up; the median follow-up was 45 (range: 0.23–154) months. The survival rates were calculated using the life table methods and presented using the Kaplan-Meier method. For statistical comparison, overall survival (OS) curves were obtained at 5 years. In addition, multivariate analysis was done for the significant factor. Cox regression, for all categorical confounders, was used and was based on explanatory modelling strategy. A significance criterion of *p* ≤ 0.05 was used in the analysis. All analyses were performed using SAS version 9.4 (SAS Institute Inc., Cary, NC).

Median survival times were compared between three grade levels; grade I (*n* = 38), grade II (*n* = 226) and grade III (*n* = 170) using univariate Log rank test. Giving the known poor outcome associated with grade III tumors, data was regrouped by combining first two grades together and re-tested against grade III.

## Results

### Demographics

A total of 553 patients were included; 12 (2.2%) were males. The mean age ± SD (71 ± 5.1), and the median is 70 (7.2) years. However, only 121 (21.9%) were 75 years or older and 40 (7.2%) were ≥ 80 years old. Family history of breast cancer in first degree relatives was identified among 115 (20.8%); 18 (15.7%) of them with an additional cancer other than breast.

### Clinicopathologic features

Invasive ductal carcinoma (IDC) was the predominant pathology identified among 460 (83.2%) while invasive lobular carcinoma (ILC) was seen among 60 (10.8%). On presentation, 114 (20.6%) patients had metastatic disease and was mostly visceral (81; 71.1%). Patients with non-metastatic disease had poor pathological features including node-positive in 244 (55.6%), grade-III in 170 (38.7%) and lymphovascular invasion in 173 (39.4%). Among the 501 patients with known HER-2 status, 92 (18.4%) were positive and 38 (7.6%) had triple-negative disease. Larger tumors with T3 and T4 disease were seen in 48 (10.9%) and 13 (3.0%), respectively, Table 1.

**Table 1:** Patients characteristics, (*n* = 553)

**Characteristics**		**Number of patients**	**Percentage**
Gender	Female	541	97.8%
	Male	12	2.2%
Age group (years)	65–69	270	48.8%
	70–74	162	29.3%
	75–79	81	14.6%
	≥ 80	40	7.2%
Histology	IDC	460	83.2%
	ILC	60	10.8%
	Others^c^	33	6.0%
Tumor Size (T)^a^	Tis T1	2 114	0.23% 26.0%
	T2	229	52.2%
	T3	48	10.9%
	T4	13	3.0%
	Tx	35	7.5%
Axillary nodal metastasis^a^	Negative	178	40.5%
	Positive	244	55.6%
	Unknown	17	3.9%
Stage	I	74	13.4%
	II	109	19.7%
	III	241	43.6%
	IV	114	20.6%
	NA	15	2.7%
Grade^a^	I	38	8.7%
	II	226	51.5%
	III	170	38.7%
	NA	5	1.1%
Lymphovascular invasion (LVI)^a^	Unidentified Negative Identified Unknown	204	47.0%
		173	39.4%
		62	14.1%
Progesterone Receptors (PR)	Negative	104	18.8%
	Positive	441	79.7%
	Unknown	8	1.4%
Estrogen Receptors (ER)	Negative	87	15.7%
	Positive	458	82.8%
	Unknown	8	1.4%
HER2-neu^b^	Negative	409	81.6%
	Positive	92	18.4%
	Unknown	52	9.4%

*NA* Not available

^a^For M0 patients *n* = 439

^b^From the 501 patients tested for HER2

^c^Include: Papillary, Medullary and Metaplastic

**Fig. 1:**
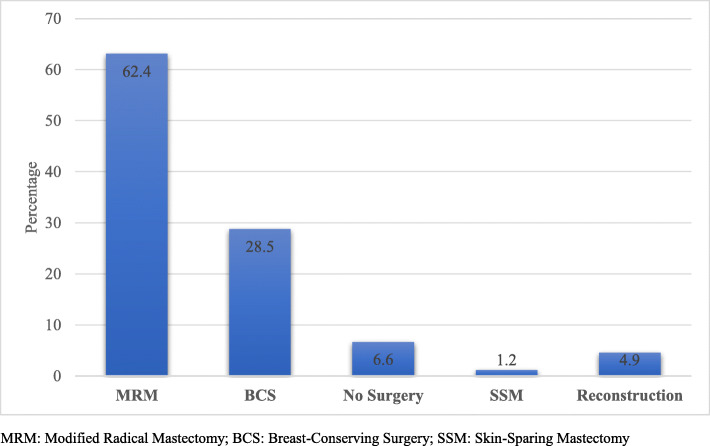
Surgical interventions for patients with non-metastatic disease (%). *Footnote: MRM: Modified Radical Mastectomy; BCS: Breast-Conserving Surgery; SSM: Skin-Sparing Mastectomy*

Among the patients with non-metastatic disease, modified radical mastectomy was the most performed surgery (274; 62.4%) while 125 (28.5%) patients had breast conserving surgery (BCS). Additionally, 29 (6.6%) never had surgery mostly because of patients’ refusal (*n* = 5), comorbidities or poor performance status (*n* = 20). Sentinel lymph node biopsy (sLN) was performed on 156 (38.0%) while axillary dissection; upfront or following a positive sLN, was performed on 285 (69.5%%). Breast reconstruction, both immediate and delayed, was performed on only 20 (4.9%) of the patients, Fig. 1.

Among patients with non-metastatic disease, 279 (63.6%) were treated with chemotherapy; 67 (24.0%) were in the neoadjuvant setting. However, 144 (32.8%) had no chemotherapy because of low-risk disease, patient refusal or poor performance status. Similarly, chemotherapy was offered for only 16 (14.0%) patients with metastatic disease. All patients, with hormone-receptor positive tumors, were treated with aromatase inhibitors.

### Survival

**Fig. 2:**
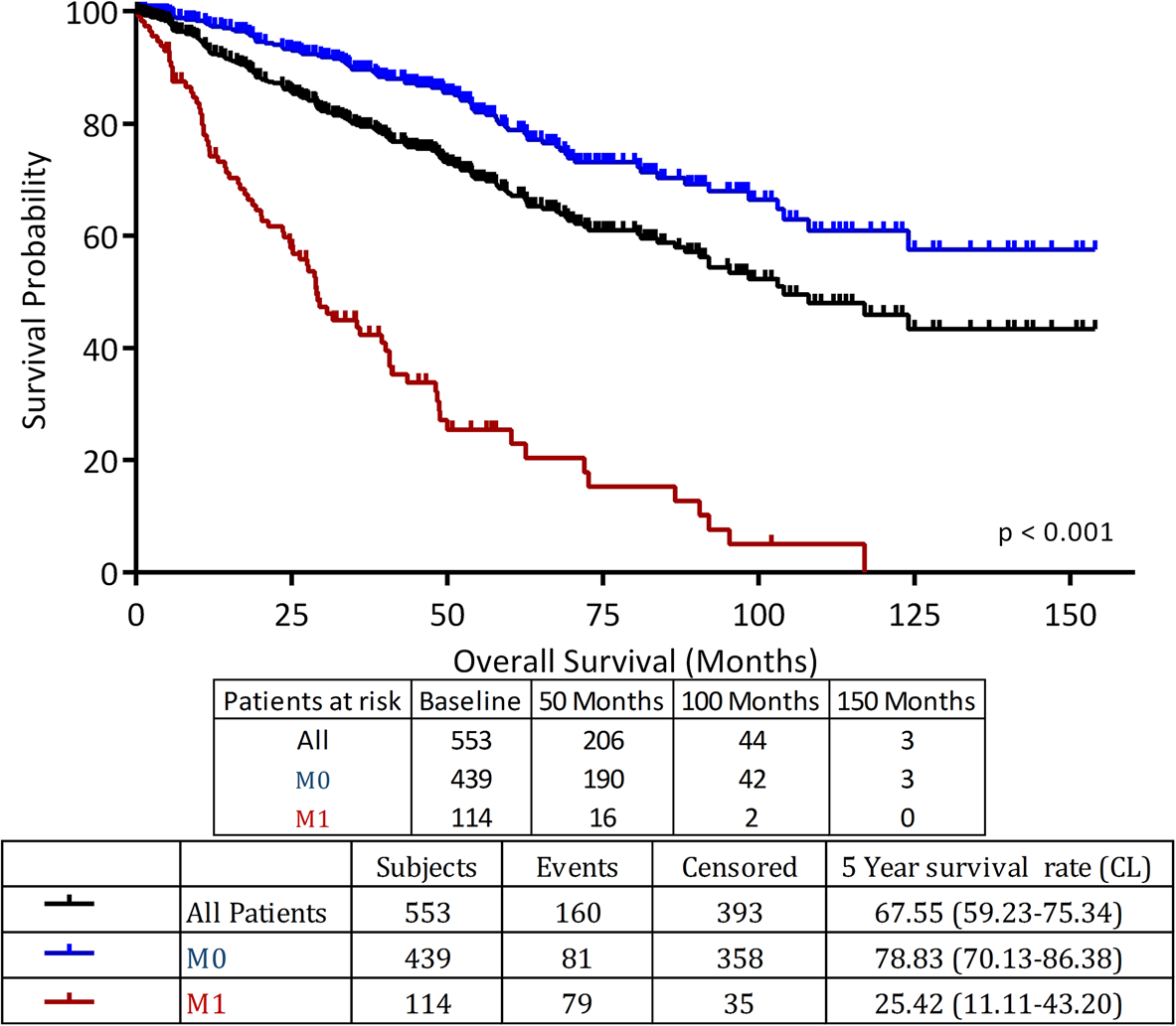
Overall survival for the whole group and by disease stage (*n* = 553)

After a median follow up of 45 (range: 0.23–154) months, 5-year overall survival for the whole group was 67.6% while the median overall survival was 104.2 months. Survival was significantly better for patients with non-metastatic disease with 5-year OS of 78.8% compared to 25.4% for patients with metastatic disease; *P* < 0.0001 (Fig. 2).

**Fig. 3:**
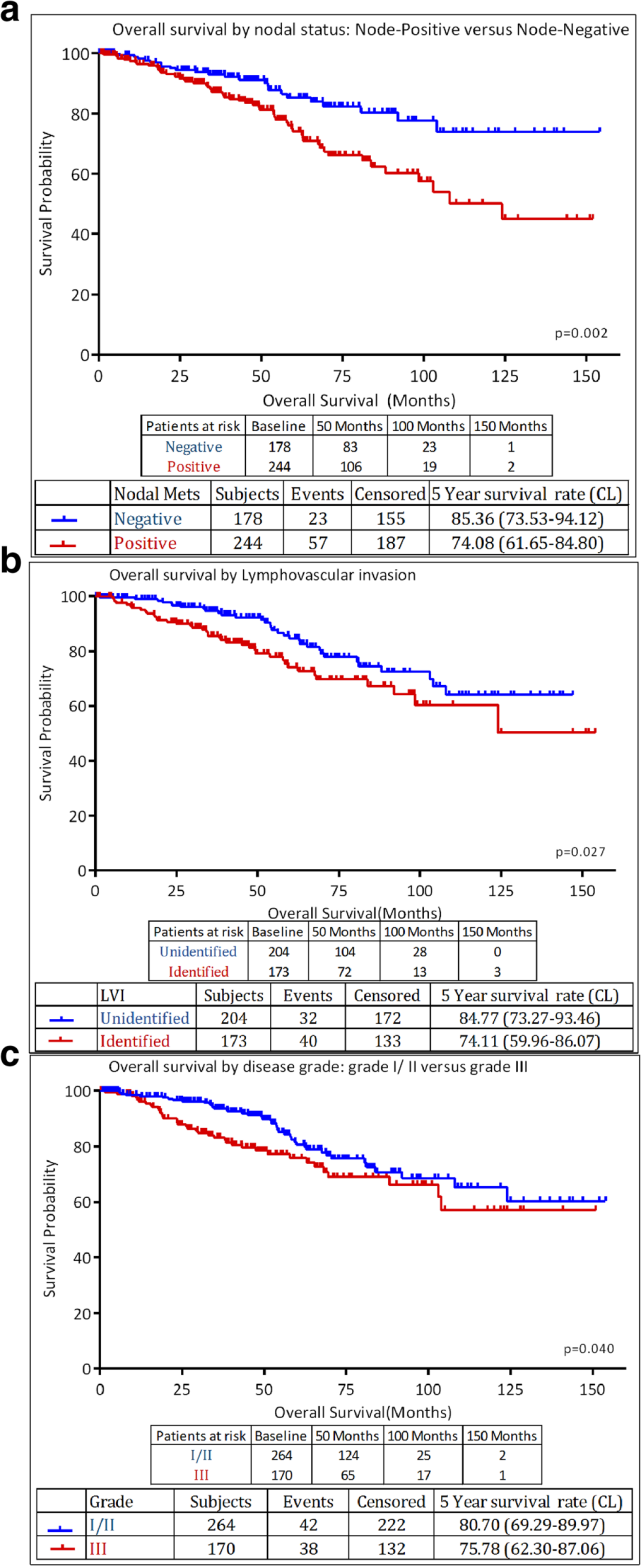
**a**: Overall survival by nodal status: Node-Positive versus Node-Negative. **b**: Overall survival by Lymphovascular invasion. **c**: Overall survival by disease grade: grade I/ II versus grade III

Among the patients with non-metastatic disease, survival was significantly better for patients with node-negative compared to those with node-positive disease; 5-year OS was 85.4 and 74.1%, respectively, *P* = 0.002 (Fig. 3a). Survival advantage was also noted among patients with no lymphovascular invasion (LVI) and those with low-grade disease as illustrated in Fig. 3b and c, respectively.

On Cox regression, only positive lymph nodes were associated with poor outcome in patients with non-metastatic disease (Hazard Ratio [HR], 1.75; 95% CI: 1.006–3.034, *P* = 0.048). Tumor grade (grade-III versus grade I and II) and LVI were not significant, Table 2.

**Table 2:** Cox Regression for patients with nonmetastatic disease (*n* = 370)

Parameter		*p*-value	Hazard Ratio	95% Hazard Ratio Confidence Limits	
Nodal metastasis	Positive vs. Negative	0.0476	1.747	1.006	3.034
Triple-Negative	Yes vs. No	0.1901	1.696	0.770	3.735
Grade	III vs. I + II	0.1595	1.407	0.874	2.264
Lymphovascular invasion (LVI)	Identified vs. Unidentified	0.3123	1.298	0.783	2.153

## Discussion

Jordan is a middle-income country with an estimated total population of 10 million, the majority of them are the younger generation, and only 3.7% are 65 years or older [15]. However, given the changing demographics and health care, this group is expanding rapidly.

Age is an important risk factor for breast cancer. However, data on whether patients’ age at diagnosis is also related to breast cancer treatment outcomes and survival in our region is lacking. Life expectancy for Jordanian females is significantly lower compared to Western societies [16].

Our data presented in this paper shows that chemotherapy and surgery were not aggressively used to treat a significant proportion of our patients, especially those with metastatic disease. Less than two-thirds of those with non-metastatic disease and only 14% of those with metastatic disease received chemotherapy. Similarly, surgical interventions were less aggressive. Less than a third had BCS while sLN biopsy was performed on 38.0% and axillary dissection was performed less often than younger patients [17]. Though breast reconstructive surgery is not commonly performed in our region, less than 5% of our older patients included in this study had it.

Avoidance of both surgery and chemotherapy in this age group was also reported in Western literature [18]. Hormonal therapy use as the sole therapy for breast cancer increases with age. One study at UK hospitals showed an increase from 2.8% in patients aged 65–69 years to 40.3% among patients aged 70 years or older [19]. Furthermore, it has been shown in previous studies that older women are less likely to receive adjuvant radiotherapy [4, 20, 21].

Variation in the rate of surgery for breast cancer persists even in the same hospital. In one study that utilized data on over 17,000 women aged 70 years or more with ER-positive operable breast cancer from two UK regional cancer registries demonstrated considerable variation in rates of surgery. Despite adjusting for case-mix, this variation persisted at the hospital level [10]. Utilizing the Charlson’s Index of co-morbidity, Giordano and colleagues reported that among women aged 75 years or older treated for breast cancer with clinical stage I-IIIa, the odds of having surgery in accordance with the guidelines were 0.32 (95% confidence interval (CI) 0.20 to 0.51) times lower than those of 55–64-year-old [22].

Because treatment decisions for such older patients are based mostly on age rather than health status or potential benefit, objective tools that assess the fitness and functional status of older patients for the planned cancer treatment is highly needed [23, 24]. A study from Sweden that included 4453 women diagnosed with breast cancer in Malmö University Hospital between 1961 and 1991 looked at the effect of age on breast cancer-specific mortality. When adjusted for potential confounders, including stage at diagnosis, age was a significant factor only for patients aged 80 years or more [25].

Based on women diagnosed with breast cancer between 2008 and 2014, the 5-year OS rate, published by the American Cancer Society, based on SEER (Surveillance, Epidemiology, and End Results)-database, for patients with stage IV disease is 27% [26]. This number had increased from 22% in 2012 [27]. The SEER database, however, does not group cancers by the American Joint Committee on Cancer (AJCC) TNM stages, instead, it groups cancers into localized, regional, and distant stages. The 5-year OS rates for patients with regional disease is 85%. Our survival rates are a little lower. However, the two populations are not comparable. Several of the known poor prognostic pathological features, like positive axillary lymph nodes and high-grade tumors are more prevalent in our patient population compared to what was reported in Western literature. The prevalence of comorbidities among our population, in general, is high enough to explain our lower life expectancy and this obviously affect the aggressiveness of anticancer therapy for this population and may be another factor to explain this difference in OS.

Our study is not without limitations. This is a retrospective study with limited data on potentially important factors like performance status, detailed comorbidities and social support. Though our study is a single-institution one, our center treats over two-thirds of all breast cancer patients in the country.

## Conclusions

Due to late presentation, older women with breast cancer in developing countries present with more aggressive features and advanced-stage disease that reflect poorly on treatment outcomes. Because of comorbidities and poor performance status, some patients are not aggressively treated. Future regional studies should focus on identifying tumor and patient-related characteristics and link it to objective measures that can be used to help target anti-cancer therapy for older patients most likely to benefit. Awareness about the need to treat “older patients” more aggressively is growing and plans to create geriatric oncology service are ongoing.

## Data Availability

Data will be made available as per the Journal and publisher rules and regulations. Readers may contact the corresponding author [HA]1 for details related to data.

## Abbreviations

HR: Hazard Ratio
HER2: Human Epidermal growth factor Receptor 2
ER: Estrogen Receptor
PR: Progesterone Receptor
SEER: Surveillance Epidemiology and End Results
IRB: Institutional Review Board
OS: Overall Survival
BCS: Breast Conserving Surgery
sLN: Sentinel lymph node
LVI: Lymphovascular Invasion
MRM: Modified Radical Mastectomy
SSM: Skin-Sparing Mastectomy
